# The Interplay Between Policy and COVID-19 Outbreaks in South Asia: Longitudinal Trend Analysis of Surveillance Data

**DOI:** 10.2196/24251

**Published:** 2021-06-17

**Authors:** Sarah B Welch, Dinushi Amanda Kulasekere, P V Vara Prasad, Charles B Moss, Robert Leo Murphy, Chad J Achenbach, Michael G Ison, Danielle Resnick, Lauren Singh, Janine White, Tariq Z Issa, Kasen Culler, Michael J Boctor, Maryann Mason, James Francis Oehmke, Joshua Marco Mitchell Faber, Lori Ann Post

**Affiliations:** 1 Buehler Center for Health Policy & Economics Feinberg School of Medicine Northwestern University Chicago, IL United States; 2 Feinberg School of Medicine Northwestern University Chicago, IL United States; 3 Sustainable Intensification Innovation Lab Department of Crop Ecophysiology Kansas State University Manhattan, KS United States; 4 Food and Resource Economics Department University of Florida Gainesville, FL United States; 5 Divison of Infectious Diseases Feinberg School of Medicine Northwestern University Chicago, IL United States; 6 International Food Policy Research Institute Washington, DC United States

**Keywords:** 7-day lag, acceleration, Bangladesh, Bhutan, COVID-19 surveillance, COVID-19, dynamic panel data, India, jerk, Maldives, Pakistan, South Asia, speed, Sri Lanka

## Abstract

**Background:**

COVID-19 transmission rates in South Asia initially were under control when governments implemented health policies aimed at controlling the pandemic such as quarantines, travel bans, and border, business, and school closures. Governments have since relaxed public health restrictions, which resulted in significant outbreaks, shifting the global epicenter of COVID-19 to India. Ongoing systematic public health surveillance of the COVID-19 pandemic is needed to inform disease prevention policy to re-establish control over the pandemic within South Asia.

**Objective:**

This study aimed to inform public health leaders about the state of the COVID-19 pandemic, how South Asia displays differences within and among countries and other global regions, and where immediate action is needed to control the outbreaks.

**Methods:**

We extracted COVID-19 data spanning 62 days from public health registries and calculated traditional and enhanced surveillance metrics. We use an empirical difference equation to measure the daily number of cases in South Asia as a function of the prior number of cases, the level of testing, and weekly shifts in variables with a dynamic panel model that was estimated using the generalized method of moments approach by implementing the Arellano–Bond estimator in R.

**Results:**

Traditional surveillance metrics indicate that South Asian countries have an alarming outbreak, with India leading the region with 310,310 new daily cases in accordance with the 7-day moving average. Enhanced surveillance indicates that while Pakistan and Bangladesh still have a high daily number of new COVID-19 cases (n=4819 and n=3878, respectively), their speed of new infections declined from April 12-25, 2021, from 2.28 to 2.18 and 3.15 to 2.35 daily new infections per 100,000 population, respectively, which suggests that their outbreaks are decreasing and that these countries are headed in the right direction. In contrast, India’s speed of new infections per 100,000 population increased by 52% during the same period from 14.79 to 22.49 new cases per day per 100,000 population, which constitutes an increased outbreak.

**Conclusions:**

Relaxation of public health restrictions and the spread of novel variants fueled the second wave of the COVID-19 pandemic in South Asia. Public health surveillance indicates that shifts in policy and the spread of new variants correlate with a drastic expansion in the pandemic, requiring immediate action to mitigate the spread of COVID-19. Surveillance is needed to inform leaders whether policies help control the pandemic.

## Introduction

### Background

The director general of the World Health Organization officially declared the outbreak of SARS-CoV-2, the virus that causes COVID-19, a pandemic on March 11, 2020 [1]. The first cases of COVID-19 in South Asia were reported in India on January 30, 2020 [2], while Pakistan confirmed its first 2 cases on February 26, 2020 [3]. In response, leaders worldwide weighed the costs of saving lives over saving livelihoods [4-6] by implementing the “Great COVID Shutdown.” While remanding citizens to their households strained the economies in Europe, Asia, and North America, the Great COVID Shutdown had a much more profound impact on low- and middle-income countries [7]. Low-income countries were disproportionately affected as their jobs and businesses were immediately obliterated, which resulted in abrupt increases in poverty and hunger [8-10].

**Figure 1:**
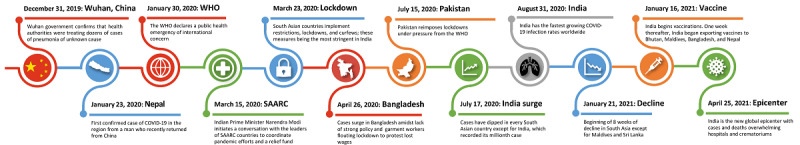
Timeline of COVID-19 events in South Asia. SAARC: South Asian Association for Regional Cooperation, WHO: World Health Organization.

The South Asian Association for Regional Cooperation, which comprises India, Pakistan, Bangladesh, Nepal, Sri Lanka, Maldives, Bhutan, and Afghanistan, met on March 15, 2020, to address the COVID-19 crisis [11,12]. South Asian countries began implementing mitigation efforts such as mask-wearing, social distancing, and closing schools and businesses in India, Bhutan, Bangladesh, and Sri Lanka in March 2020 [11]. By March 24, 2020, existing surveillance efforts in South Asia reported only 1536 cases and 22 deaths [13]. By April 2021, the number of observed COVID-19 cases and deaths in South Asian countries reached 18 million and 219,000, respectively [14], which indicated alarming growth. Figure 1 shows the timeline of COVID-19 events in South Asia.

**Figure 2:**
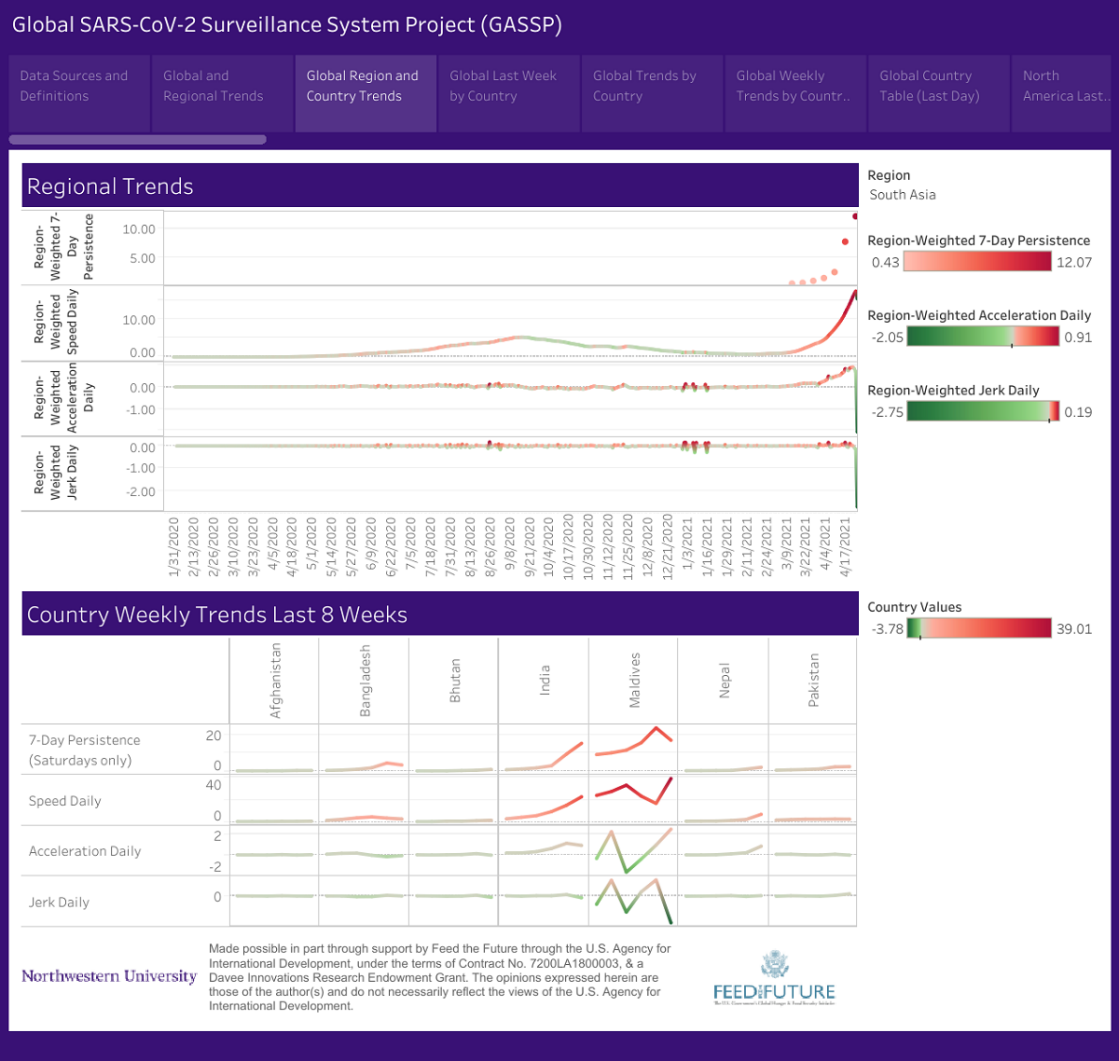
Weekly SARS-CoV-2 trends in South Asian countries (January 31, 2020 to April 25, 2021).

In general, South Asia displayed a particularly unique trajectory since the onset of the pandemic. First, the region was slow to build their caseload, particularly when compared to other global regions in Europe, Central Asia, the Middle East, and the Americas [11]. Second, the significant uptick in COVID-19 transmissions in South Asia began accruing after international border lockdown policies were implemented to stop the spread of COVID-19, which forced migrant workers from South Asia to travel back to their home countries [15]. Globally, India is the largest source of migrant workers, followed by Bangladesh in sixth position and Pakistan in seventh position; this explains why India, Bangladesh, and Pakistan were the 3 most affected countries at the beginning of the pandemic in South Asia [16]. Migrant workers produce tremendous economic benefits; however, migration accelerates human interactions, which increases the spread of the disease. While human mobility has important benefits for economic growth, migration inadvertently leads to disease spread in a low-incidence setting. The movement of migrants toward their home countries before border lockdown measures were implemented, both internationally and within countries, was the largest mass migration since the 1947 partition of India, Pakistan, and Bangladesh [17]. The fear of migrants carrying COVID-19 rippled throughout South Asia, which subsequently only served to displace COVID-19 from the host countries of migrants to their home countries [18].

By early fall of 2020, India was on track to overtake the United States in the magnitude of COVID-19 transmissions but reversed course and cases declined after the implementation of strict stay-at-home policies in addition to other COVID-19 prevention policies [19]. The government of India also initiated social assistance packages to support the agricultural sector and provided direct and indirect relief to workers and households to minimize economic impacts [20]. Although there were short-term benefits and agriculture was the only sector that displayed positive growth (3.4%) in the first quarter of 2020 [21], the long-term impact of the lockdown could be negative. By December 2020, the pandemic in the South Asian region was in decline owing to strict lockdowns and travel bans.

The easing of disease prevention policies in favor of economic, food security, social, and political concerns [22,23] along with the new SARS-CoV-2 strains or variants [24], resulted in explosive growth of the COVID-19 pandemic in early 2021, with India surpassing the United States’ single-day records [25-29], thus overwhelming the health care system [30]. While global leaders must weigh policies that are aimed at protecting livelihoods, they also must consider their impact on lives and should be informed by ongoing systematic public health surveillance.

### Objective

Our objective is to use COVID-19 surveillance to inform policy decisions regarding the pandemic; it is important to understand how South Asia differs between and within countries and from other global regions and to pinpoint where immediate action is needed to control the outbreaks. To that end, in addition to traditional surveillance metrics, we used dynamic panel modeling and the generalized method of moments, which correct for limitations in existing surveillance. Parallel work utilizing enhanced surveillance metrics has been completed for sub-Saharan Africa [31], the United States [32], the Middle East and North Africa [33], Central Asia [34], Europe [35], Latin America and the Caribbean [36], East Asia and the Pacific [37], Canada [38], and metropolitan regions [39].

## Methods

Using application programming interfaces, we automatically extract data daily from the Foundation for Innovative New Diagnostics [40]. The Foundation for Innovative New Diagnostics compiles data from multiple sources across individual websites, statistical reports, and press releases. We also accessed data for the most recent 8 weeks from the GitHub repository [41]. This resulted in a panel of 8 countries with 62 days in each panel (n=496). We calculated traditional surveillance indicators as specified below. In addition, we used enhanced surveillance metrics, which perform better at measuring the dynamics of the pandemic and control for data contamination by using an empirical difference equation. We specified the number of new positive cases each day in each country as a function of the prior day’s number of new cases, the number of new cases 7 days prior, the level of testing, and weekly shifts in variables that help determine whether the pandemic is expanding or contracting compared to prior weeks. This resulted in a dynamic panel model estimated using the generalized method of moments approach implementing the Arellano–Bond estimator in R [42,43].

Traditional surveillance indicators include the total number of cases and deaths, the 7-day moving average of new cases, and the 7-day moving average of deaths. Enhanced surveillance metrics [44,45] include the following: (1) speed: the weekly average number of new positive tests per day divided by the total country population and multiplied by 100,000; (2) acceleration: the weekly average of daily changes in the speed of the infection; (3) jerk: the change in the acceleration of the infection; and (4) the 7-day persistence effect on speed, which refers to the number of new cases reported on the present day, which are statistically attributable to new cases reported 7 days ago.

## Results

### Country-Specific Results of Regression Analysis

We grouped 8 countries including Afghanistan, Bangladesh, Bhutan, India, Maldives, Nepal, Pakistan, and Sri Lanka in South Asia and present the results of regression analysis in Table 1. The weekly surveillance products are a function of these regressions.

The regression Wald statistic was significant (χ^2^_6_=10509826; *P*<.001). The Sargan test revealed nonsignificant findings, failing to reject the validity of overidentifying restrictions (χ^2^_513_=8; *P*>.99).

**Table 1:** Arellano–Bond dynamic panel data model of COVID-19 dynamics at the country level.

Variable	Coefficient	*P* value
7-day lag	0.424	<.001
7-day lag shift	0.617	.05
Wald statistic for regression, χ^2^_6_	10509826	<.001
Sargan statistic for validity, χ^2^_513_	8	>.99

### Interpretation: Results of Regression Analysis for South Asia

The regression analyses indicate that we have a balanced panel and that we do not have overidentifying restrictions, which implies that this model fits the data. Further, regression analyses indicate a positive shift in the pandemic between April 12-18 and April 19-25, 2021.

### Surveillance Results

Traditional and enhanced surveillance results for April 12-18, 2021, are presented in Table 2 and those for April 19-25, 2021, are presented in Table 3. Data of 5 prior weeks of COVID-19 surveillance trends are provided in Multimedia Appendix 1. Data dating back to the onset of the initial cases of COVID-19 in South Asia (January 2020), as well as other regions worldwide, are provided in our active surveillance system.

**Table 2:** Surveillance metrics for the week of April 12-18, 2021.

Country	7-day moving average of new cases	Total cases	7-day moving average of new deaths	Total deaths	Daily speed	Daily acceleration	Daily jerk	7-day persistence
Afghanistan	82	57,721	3	2539	0.21	–0.01	–0.01	0.21
Bangladesh	5188	715,252	89	10,283	3.15	–0.16	0.10	4.40
Bhutan	6	952	0	1	0.78	0.13	0.11	0.37
India	204,171	14,788,003	1125	177,150	14.79	1.12	0.20	9.41
Maldives	89	26,145	0	69	16.41	0.95	2.27	24.03
Nepal	562	283,658	5	3075	1.93	0.21	–0.06	1.00
Pakistan	5038	756,285	114	16,243	2.28	0.07	0.08	2.23
Sri Lanka	215	96,354	3	615	1.00	–0.08	–0.03	N/A^a^

^a^N/A: not applicable.

**Table 3:** Surveillance metrics for the week of April 19-25, 2021.

Country	7-day moving average of new cases	Total cases	7-day moving average of new deaths	Total deaths	Daily speed	Daily acceleration	Daily jerk	7-day persistence
Afghanistan	144	58,730	5	2572	0.37	0.03	0.04	0.22
Bangladesh	3878	742,400	96	10,952	2.35	–0.07	0.00	3.28
Bhutan	9	1018	0	1	1.22	–0.02	–0.19	0.81
India	310,310	16,960,172	2166	192,311	22.49	0.91	–0.25	15.41
Maldives	211	27,621	0	71	39.01	2.48	1.53	17.09
Nepal	1918	297,087	9	3136	6.58	0.84	0.05	2.01
Pakistan	4819	790,016	108	16,999	2.18	–0.01	–0.08	2.38
Sri Lanka	605	100,586	3	638	2.82	0.60	0.23	N/A^a^

^a^N/A: not applicable.

Overall, South Asia saw cases increasing steadily during the second wave of infections from March 8 to April 25, 2021, after steady declines in January and February 2021; however, country-level subanalysis with enhanced surveillance indicates that there are significant differences among South Asian countries (Multimedia Appendix 1).

In India, the 7-day moving average of daily new cases increased from 204,171 in the week of April 12-18, 2021, to 310,310 in the week of April 19-25, 2021, while the 7-day moving average of daily deaths increased from 1125 to 2166 per day during the same period, which indicates a 52% increase in new COVID-19 cases and a 92.5% increase in deaths per day. The speed increased from 14.79 new cases per day per 100,000 population in the week of April 12-18, 2021, to 22.49 new cases per day per 100,000 population in the week of April 19-25, 2021. An acceleration of 0.91 for the week of April 19-25, 2021, implies that the number of cases in India per 100,000 population is increasing by almost 1 case per day, although this rate is slightly lower than the acceleration of 1.12 reported in the prior week. Positive acceleration suggests the possibility of there being even more daily cases in the week of April 26-May 2, 2021, than in the week of April 19-25, 2021. The negative jerk indicates that although the rate of acceleration is decreasing, the acceleration remains positive. The reduction in India’s case rate does not imply that the pandemic is receding, but rather only that the rate of explosive growth has slightly decreased. The 7-day persistence increased from 9.41 in the prior week to 15.41, which indicates that more than two-third of the new cases in the week of April 19-25, 2021, are statistically attributable to new cases in the prior week. This indicates a context of high transmissibility, which could be caused by a combination of policy shifts, superspreader events, or the presence of more contagious variants. India is now the epicenter of the COVID-19 pandemic [46], and indicators suggest that the pandemic will continue to worsen in India in the immediate future.

Maldives saw an increase in the 7-day moving average number of daily new COVID-19 transmissions from 89 to 211 and 0 new deaths between the weeks of April 12-18 and April 19-25, 2021. While this is an increase of 133%, the absolute caseload is small. The daily speed increased from 16.41 per 100,000 population in the week of April 12-18, 2021, to 39.01 per 100,000 population in the week of April 19-25, 2021, with an acceleration of 0.95 during the week of April 12-18, 2021, and a more rapid acceleration of 2.48 per 100,000 population during the week of April 19-25, 2021. The positive jerk of 1.53 indicates the increase in acceleration in addition to the speed at which the pandemic is expanding within Maldives. The 7-day persistence rate of 17.09 indicates that 44% of the new cases in the week of April 19-25, 2021, are statistically attributable to new cases in the prior week (ie, April 12-18, 2021). This indicates a context of high transmissibility; however, fewer cases are echoing forward.

Afghanistan saw a slight expansion in the pandemic from the week of April 12-18 to April 19-25, 2021, with an increase in speed from 0.21 to 0.37 per 100,000 population, and a shift from deceleration to an acceleration in pandemic growth of 0.03. This indicates a rising number of cases and increasing acceleration. The 7-day persistence of 0.22 in the week of April 19-25, 2021, compared to 0.21 in the week of April 12-18, 2021, indicates that new cases are statistically linked to cases in the prior week. The 7-day moving average of daily new cases increased from 82 to 144 over the same period. The ongoing growth is not ideal but does not signal alarming growth in the pandemic at this point.

Bangladesh saw a slight reduction in the pandemic from the week of April 12-18 to April 19-25, 2021, with a reduction in speed from 3.15 to 2.35 per 100,000 population, along with sustained deceleration. The 7-day moving average of daily new cases decreased from 5188 to 3878 cases between the weeks of April 12-18 to April 19-25, 2021. The 7-day persistence of 3.28 in the week of April 19-25, 2021, indicates that the cases this week are linked to those in the prior week and that the rate at which the cases are echoing forward is decreasing. Bangladesh is moving in the right direction.

Bhutan saw a slight expansion in the pandemic from the week of April 12-18 to April 19-25, 2021, with an increase in speed from 0.78 to 1.22 cases per 100,000 population; however, there was a shift from an acceleration of 0.13 to a deceleration –0.02. The 7-day persistence of 0.81 in the week of April 19-25, 2021, indicates that 46% of new transmissions are statistically linked to cases in the prior week. Since the caseload is small with 7-day averages of 6 new daily cases the week of April 12-18, 2021, increasing to 9 new cases in the week of April 19-25, 2021, Bhutan’s current condition is less concerning than that of other countries in the region.

Nepal’s speed is much lower than that of India or Maldives, but its daily speed of 6.58 new cases per 100,000 population in the week of April 19-25, 2021, is much higher than that of other countries in the region. This is an alarming increase in the daily speed from 1.93 per 100,000 population in the prior week for an increase in the 7-day moving average of new cases from 562 to 1918 per 100,000 population, a 3.4-fold increase in new transmissions. Acceleration increased from 0.21 in the week of April 12-18, 2021, to 0.84 in the week of April 19-25, 2021, indicating an increase in the rate of the pandemic. The 7-day persistence of 2.01 in the week of April 19-25, 2021, indicates that 50% of new cases are statistically linked to cases in the prior week. These indicators signal an alarming growth in Nepal.

Pakistan saw a very slight reduction in the pandemic from the week of April 12-18 to April 19-25, 2021, with a small reduction in speed from 2.28 to 2.18 cases per 100,000 population, and a shift from positive acceleration to deceleration in the pandemic. Pakistan’s 7-day moving average of daily new cases slightly decreased from 5038 to 4819 over the 2-week period. The 7-day persistence of 2.38 in the week of April 19-25, 2021, indicates that approximately 94% of cases are statistically linked to cases in the prior week. The increase in persistence suggests an increasing clustering effect. Surveillance indicates that the pandemic is declining, but attention must be paid to the 7-day persistence as echoing cases are problematic, and more cases are echoing forward in Pakistan.

Sri Lanka, like Nepal, showed alarming growth in the pandemic from the week of April 12-18 to April 19-25, 2021, with an increase in speed from 1.00 to 2.82 cases per 100,000 population, which indicates a shift from a deceleration of –0.08 to an acceleration of 0.60 and a positive jerk. The necessary data needed to calculate the 7-day persistence are missing for Sri Lanka, but available surveillance measures indicate alarming growth.

## Discussion

### Principal Findings

Surveillance efforts have the capacity to inform leaders when policies are effective (and when they are not) [47] and are the foundation of public health [48]. However, for surveillance data to be most useful and actionable, they must be contextualized by the population and characteristics of the country in question. The most populous countries in South Asia are India, Pakistan, and Bangladesh. India is >6-fold larger than the next largest country in the region, Pakistan, and has an approximately 3-fold larger population than that of all other countries in the region combined. To that end, significant increases in the caseload and rates of transmission are alarming in a country with more than 1.3 billion people. In contrast, Bhutan has <1 million residents; hence, the speed of the pandemic is less concerning when the absolute number of cases are in single digits.

Traditional surveillance measures are useful but not sufficient to capture these distinctions, nor do they describe the dynamics of the pandemic. India and Maldives present a perfect case study with respect to the importance of using novel surveillance metrics. While India is more newsworthy because it ranks #1 in the absolute number of new COVID-19 cases, since its total population exceeds 1.3 billion people, the outbreak in Maldives is worse when controlling for population size. Explosive growth of the pandemic in Maldives does not affect as many individuals nor poses the same threat globally, as is the case of the outbreak in India; however, it is important to use the surveillance measures to identify the grave public health threat this outbreak poses to residents in Maldives and not let its relatively small size become overshadowed. The identification of concerning outbreaks, so they can be stymied, is imperative for the health of local regions.

Standard surveillance efforts provide a proxy for caseload (owing to asymptomatic and untested COVID-19–positive persons), which are helpful to understand the current levels of COVID-19 outbreaks; however, enhanced surveillance indicates how fast the outbreak is expanding and the alarming rates of explosive growth, as is the case in India. Speed, acceleration, jerk, and persistence are helpful metrics to compare the condition in each South Asian country relative to or within the region and to identify the need for sustaining actions or new interventions that lead to a reduction in the pandemic.

COVID-19 surveillance during the week of April 12-25, 2021, indicates that with the exception of Bangladesh and Pakistan, South Asia is experiencing an alarming outbreak of COVID-19. First, while there are many similarities in terms of development, the environment, and population structure, South Asian countries are heterogeneous in terms of their culture, economies, and susceptibility to the COVID-19 pandemic [49]. Second, migrant workers are central to disease control in South Asia, considering the large number of migrants in most South Asian countries [50]. Relaxing of the travel ban and reopening of borders have increased human interaction as migrants have passed through borders and used overcrowded public transportation to travel to their host countries, which has further fueled the COVID-19 outbreaks [18]. When South Asian leaders relaxed COVID-19 restrictions to reopen the economy [22], the pandemic markedly resurged [51] especially in India and South Asian island nations. Third, new variants B.1.1.7 (Alpha) and B.1.617.2 (Delta) harbor mutations that may have contributed to the significant impact of the current wave of COVID-19 in most South Asian countries [51]. The use of surveillance data to inform policy-level action to combat this outbreak must consider these local contexts.

Unfortunately, South Asia has experienced 2 waves of COVID-19, and all 8 countries still have active new transmissions on a daily basis, which leads to questions such as, “how will public health leaders know when the pandemic is receding?” or “are policies put in place to control the pandemic working?” Our findings indicate that adding enhanced surveillance to standard surveillance including measures of speed, acceleration, jerk, and persistence, can help to answer such questions. Additionally, enhanced surveillance metrics can help track pandemic growth factors that in turn help identify areas of concerning growth. While South Asia reversed its trajectory of the COVID-19 pandemic in late 2020 and early 2021, this trend decelerated and reversed course again, resulting in explosive growth in several South Asian countries, with Maldives and India experiencing the worst outbreaks.

By linking policies to the pandemic through systematic surveillance, it is evident when shifts result in outbreaks. Furthermore, surveillance informs public health leaders when it is safe to reopen the economy or change policy, thus establishing a delicate balance between saving lives while saving livelihoods. Undoubtedly, policymakers are faced with difficult decisions in their attempts to balance the health of their residents with the highly credible economic threat posed by the COVID-19 pandemic. It is estimated that an additional 75 million people fell below the poverty line owing to the COVID-19 pandemic in 2020, accounting for approximately 60% of the global increase in poverty in 2020 [52]. The dichotomy of lives versus livelihood may seem complex, but if people are alive, livelihoods can be addressed through appropriate policies. Dual focus on both improving pandemic surveillance and the needs of the health care system for protecting lives, and ensuring social protection for the agricultural sector and smallholder farmers for increased resilience, food, and nutritional security are needed.

COVID-19 cases had been decreasing steadily in South Asia since they peaked in late September 2020, but increased public gatherings and relaxing of public health restrictions led to the latest surge [53]. It is likely that currently implemented public health policies to stop the spread of COVID-19 were relaxed too soon after experiencing several weeks of decline in the number of active cases. For example, opening borders to allow migrant workers to travel back and forth between their host and home countries corresponded with an outbreak. Ideally, leaders should wait until the speed, acceleration, and jerk reach 0 before changing the policies on border openings, followed by careful surveillance to ensure that no hidden pockets of COVID-19 magnify into an outbreak. Alternatively, a staged approach to reopening is more realistic with subnational analysis for larger countries. For example, in areas where COVID-19 transmissions have stopped, allowing businesses to reopen to address economic concerns but banning travel to and from locations with active COVID-19 cases would result in fewer transmissions than a blanket retraction of all policies.

### Limitations

Our findings are limited by the granularity in the country-level data. Data are reported on a national level for countries within South Asia, which precludes intranational analyses that would more closely reflect local regulations and better contextualize national trends. In addition, suboptimal public health infrastructure prevents data from being reported instantaneously and has severely limited the completeness of data from Sri Lanka. Multiple-day data are frequently collated into a single report, which may suggest almost 0 infections or deaths over a period of days, followed by a sudden spike in those same measures. Our analysis partially addresses this issue by calculating 7-day averages per 100,000 population for all metrics.

### Comparison With Prior Work

We conducted similar studies based on dynamic panel data derived from other global regions [31-39,44,45] similar to South Asia.

### Conclusions

The 7-day average number of cases and deaths, speed, acceleration, and persistence of new COVID-19 cases reveals an alarming outbreak in India and Maldives and concerning outbreaks in Afghanistan, Bhutan, Nepal, and Sri Lanka. While Bangladesh and Pakistan still have high daily caseloads, enhanced surveillance metrics indicate a reduction in their rates of novel infections. Because their daily caseload remains high, Bangladesh and Pakistan must remain vigilant in maintaining public health guidelines that will prevent additional outbreaks. Currently, these 2 countries are headed in the right direction.

While the United States still has the highest number of cumulative COVID-19 cases worldwide [32,54], the reality is that the United States only comprises 4% of the global population, while South Asia comprises 25% of the global population. India will fare much worse than the United States if the country fails to implement policies to mitigate the spread of COVID-19 or if public health restrictions in India remain as low as in 2020. Given the size of the region, prior and current outbreaks, a paucity of vaccines, and relaxed public health guidelines, South Asia in general, and India in particular, are set to exceed the United States’ total caseload and deaths in the absence of massive intervention. The fact that India is surpassing the United States’ daily record of new COVID-19 cases, combined with metrics including the pandemics’ speed, acceleration, jerk, and persistence, indicates that India’s alarming outbreak will only worsen. The current outbreak poses an immediate threat not only to the region, but also worldwide. Alarming outbreaks in South Asia are risky to any other region or country as COVID-19 can resurge and result in further outbreaks. The pandemic will not end anywhere until it ends everywhere [55].

## Appendix

Multimedia Appendix 1 Tables S1-S5.
