# Supplementary material for: The Interplay Between Policy and COVID-19 Outbreaks in South Asia: Longitudinal Trend Analysis of Surveillance Data
Source: JMIR Public Health Surveill. 2021 Jun 17;7(6):e24251. doi: 10.2196/24251 (PMC8213065; doi:10.2196/24251)
Supplement: Multimedia Appendix 1 [file publichealth_v7i6e24251_app1.docx]

**Table S1. Surveillance Metrics for the Week of 3/8/21 – 3/14/21**

| **Country** | New Cases 7-Day Moving Average | Total Cases | New Deaths 7-Day Moving Average | Total Deaths | Speed Daily | Acceleration Daily | Jerk Daily | 7-Day Persistence |
| --- | --- | --- | --- | --- | --- | --- | --- | --- |
| Afghanistan | 17 | 55,959 | 1 | 2,454 | 0.04 | -0.00 | -0.00 | 0.02 |
| Bangladesh | 930 | 556,236 | 11 | 8,527 | 0.56 | 0.04 | 0.00 | 0.14 |
| Bhutan | 0 | 868 | 0 | 1 | 0.00 | -0.02 | -0.02 | 0.01 |
| India | 21,178 | 11,359,048 | 122 | 158,607 | 1.53 | 0.07 | -0.00 | 0.50 |
| Maldives | 116 | 21,476 | 0 | 64 | 21.49 | -0.11 | 0.00 | 11.95 |
| Nepal | 75 | 275,178 | 1 | 3,014 | 0.26 | 0.01 | 0.04 | 0.12 |
| Pakistan | 2,099 | 605,200 | 43 | 13,508 | 0.95 | 0.06 | 0.02 | 0.29 |
| Sri Lanka | 350 | 87,600 | 5 | 526 | 1.63 | 0.08 | 0.12 |  |

**Table S2. Surveillance Metrics for the Week of 3/15/21 – 3/21/21**

| **Country** | New Cases 7-Day Moving Average | Total Cases | New Deaths 7-Day Moving Average | Total Deaths | Speed Daily | Acceleration Daily | Jerk Daily | 7-Day Persistence |
| --- | --- | --- | --- | --- | --- | --- | --- | --- |
| Afghanistan | 19 | 56,093 | 1 | 2,462 | 0.05 | 0.01 | 0.02 | 0.02 |
| Bangladesh | 1,781 | 568,706 | 20 | 8,668 | 1.08 | 0.07 | 0.00 | 0.24 |
| Bhutan | 0 | 869 | 0 | 1 | 0.02 | 0.02 | 0.02 | 0.00 |
| India | 34,297 | 11,599,130 | 164 | 159,755 | 2.49 | 0.19 | 0.03 | 0.65 |
| Maldives | 128 | 22,373 | 0 | 65 | 23.71 | -0.34 | -1.16 | 9.11 |
| Nepal | 93 | 275,829 | 0 | 3,016 | 0.32 | 0.01 | -0.03 | 0.11 |
| Pakistan | 3,086 | 626,802 | 48 | 13,843 | 1.40 | 0.06 | -0.03 | 0.40 |
| Sri Lanka | 294 | 89,655 | 3 | 544 | 1.37 | -0.10 | -0.22 |  |

**Table S3. Surveillance Metrics for the Week of 3/22/21 - 3/28/21**

| **Country** | New Cases 7-Day Moving Average | Total Cases | New Deaths 7-Day Moving Average | Total Deaths | Speed Daily | Acceleration Daily | Jerk Daily | 7-Day Persistence |
| --- | --- | --- | --- | --- | --- | --- | --- | --- |
| Afghanistan | 28 | 56,290 | 1 | 2,469 | 0.07 | 0.00 | 0.00 | 0.02 |
| Bangladesh | 3,300 | 591,806 | 29 | 8,869 | 2.00 | 0.16 | -0.00 | 0.46 |
| Bhutan | 0 | 871 | 0 | 1 | 0.04 | 0.00 | 0.00 | 0.01 |
| India | 53,213 | 11,971,624 | 257 | 161,552 | 3.86 | 0.20 | -0.03 | 1.05 |
| Maldives | 147 | 23,403 | 0 | 66 | 27.22 | 2.25 | 2.22 | 10.06 |
| Nepal | 132 | 276,750 | 2 | 3,027 | 0.45 | 0.00 | -0.01 | 0.14 |
| Pakistan | 3,970 | 654,591 | 53 | 14,215 | 1.80 | 0.07 | 0.03 | 0.59 |
| Sri Lanka | 312 | 91,839 | 2 | 558 | 1.46 | 0.08 | 0.21 |  |

**Table S4. Surveillance Metrics for the Week of 3/29/21 - 4/4/21**

| **Country** | New Cases 7-Day Moving Average | Total Cases | New Deaths 7-Day Moving Average | Total Deaths | Speed Daily | Acceleration Daily | Jerk Daily | 7-Day Persistence |
| --- | --- | --- | --- | --- | --- | --- | --- | --- |
| Afghanistan | 44 | 56,595 | 4 | 2,496 | 0.11 | -0.00 | -0.01 | 0.03 |
| Bangladesh | 5,496 | 630,277 | 49 | 9,213 | 3.34 | 0.17 | -0.09 | 0.85 |
| Bhutan | 3 | 891 | 0 | 1 | 0.37 | 0.02 | -0.04 | 0.02 |
| India | 73,412 | 12,485,509 | 439 | 164,623 | 5.32 | 0.32 | 0.04 | 1.64 |
| Maldives | 178 | 24,651 | 0 | 67 | 32.98 | -1.66 | -2.27 | 11.55 |
| Nepal | 145 | 277,768 | 1 | 3,032 | 0.50 | 0.02 | 0.01 | 0.19 |
| Pakistan | 4,760 | 687,908 | 80 | 14,778 | 2.15 | 0.02 | -0.00 | 0.76 |
| Sri Lanka | 208 | 93,295 | 2 | 575 | 0.97 | -0.07 | -0.03 |  |

**Table S5. Surveillance Metrics for the Week of 4/4/21 – 4/11/21**

| **Country** | New Cases 7-Day Moving Average | Total Cases | New Deaths 7-Day Moving Average | Total Deaths | Speed Daily | Acceleration Daily | Jerk Daily | 7-Day Persistence |
| --- | --- | --- | --- | --- | --- | --- | --- | --- |
| Afghanistan | 78 | 57,144 | 4 | 2,521 | 0.20 | 0.04 | 0.03 | 0.05 |
| Bangladesh | 6,951 | 678,937 | 64 | 9,661 | 4.22 | -0.03 | -0.08 | 1.72 |
| Bhutan | 3 | 910 | 0 | 1 | 0.35 | 0.04 | -0.02 | 0.18 |
| India | 124,757 | 13,358,805 | 665 | 169,275 | 9.04 | 0.62 | 0.03 | 2.85 |
| Maldives | 125 | 25,524 | 0 | 67 | 23.07 | -0.40 | 0.58 | 15.67 |
| Nepal | 280 | 279,725 | 1 | 3,039 | 0.96 | 0.10 | 0.05 | 0.25 |
| Pakistan | 4,730 | 721,018 | 95 | 15,443 | 2.14 | 0.00 | -0.02 | 1.11 |
| Sri Lanka | 222 | 94,848 | 3 | 595 | 1.04 | 0.08 | 0.01 |  |
